# The Likelihood of Recent Record Warmth

**DOI:** 10.1038/srep19831

**Published:** 2016-01-25

**Authors:** Michael E. Mann, Stefan Rahmstorf, Byron A. Steinman, Martin Tingley, Sonya K. Miller

**Affiliations:** 1 Department of Meteorology, The Pennsylvania State University, 503 Walker Building, University Park, PA 16802; 2 Earth System Analysis, Potsdam Institute for Climate Impact Research P.O. Box 60 12 03, 14412 Potsdam, Germany; 3 Large Lakes Observatory, Department of Earth and Environmental Sciences University of Minnesota Duluth, 2205 E. 5th Street RLB 205, Duluth, MN 55812-2496; 4 Departments of Meteorology and Statistics, The Pennsylvania State University, 503 Walker Building, University Park, PA 16802.

## Abstract

2014 was nominally the warmest year on record for both the globe and northern hemisphere based on historical records spanning the past one and a half centuries^1,2^. It was the latest in a recent run of record temperatures spanning the past decade and a half. Press accounts reported odds as low as one-in-650 million that the observed run of global temperature records would be expected to occur in the absence of human-caused global warming. Press reports notwithstanding, the question of how likely observed temperature records may have have been both with and without human influence is interesting in its own right. Here we attempt to address that question using a semi-empirical approach that combines the latest (CMIP5^3^) climate model simulations with observations of global and hemispheric mean temperature. We find that individual record years and the observed runs of record-setting temperatures were extremely unlikely to have occurred in the absence of human-caused climate change, though not nearly as unlikely as press reports have suggested. These same record temperatures were, by contrast, quite *likely* to have occurred in the *presence* of anthropogenic climate forcing.

The year 1998 set a new temperature record by a large margin for both the globe and the northern hemisphere (NH). The 1998 record was matched or exceeded in 2005 and again in 2010. The precise ranks of individual years depends on both the target (e.g. NH or global mean temperature) and the particular temperature assessment (e.g. NASA ‘GISTEMP’^1^ vs. UK Met Office ‘HadCRUT4’^2^,^3^,^4^). However, 2014 set yet a new record for both the globe and the northern hemisphere in all major assessments. In the wake of the 2014 record, press accounts reported the odds of the observed run of global temperature records as being anywhere from one-in-27 million (for 13 of the 15 warmest years having occurred since 2000—see Salon.com, 1/23/15) to one-in-650 million (for 9 of the 10 warmest years having occurred since 2000—see AP, 1/16/15).

The difficulty in quantifying the rarity of the current streak of warm years is that the time series display serial correlation; the years are not independent of one another. Sources of natural variability in temperature both internal (unforced random or chaotic variability) and external (radiatively forced changes due to volcanic eruptions and variations in solar irradiance) to the climate system lead to correlation between neighboring annual mean temperature values. Such correlation leads, in turn, to reduced effective degrees of freedom in the temperature time series.

It is critical to take into account these contributions in estimating the likelihood of record temperature values. One body of past work^5^,^6^,^7^ has employed model-based fingerprint detection methods to study temperature extremes in a generic sense, though without any focus on the types of questions posed here (i.e. the likelihoods of specific observed runs of record warmth). In this approach, natural variability is estimated from the climate models themselves, which means that assessments of the likelihood of extremes is dependent on the models producing realistic natural variability. Another past study^8^ estimated the parameters of statistical noise models directly from the instrumental temperature record, without the use of information from climate models. Not accounting for the impact of anthropogenic climate change on surface temperatures, however, could yield biased estimates of the noise parameters (e.g. by overestimating the apparent degree of natural persistence and, hence, the likelihoods of sequences of rare events). Moreover, such an approach cannot distinguish between the influence of forced and purely internal natural variability. Here, we instead use a semi-empirical method that combines the most recent (CMIP5)^9^ multimodel suite of climate model simulations with observational temperature data to estimate the noise characteristics of global and hemispheric temperature variabilty.

We represent global and hemispheric mean temperature variations through a statistical model of the form,


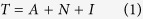


where *A* represents the anthropogenic-forced component of temperature change, *N* represents the natural (volcanic + solar) forced component of temperature change, *F* = *A* + *N* is the total forced response, and *I* represents the internal variability component (often called “noise”). Assuming that the observations provide us with *T*, while climate model simulations provide us with an estimate of *A* and *N,* the difference *T* − (*A* + *N*), i.e. the residual, can be interpreted as representing a single realization of the pure internal variability component (*I*) (a recent analysis^10^ shows that noise series estimated in this way are consistent with the more pristine noise series diagnosed from control model simulations). It is appropriate to define a stationary stochastic time series model for *I* using parameters estimated from the residual series. We perform tests to insure the adequacy of the resulting statistical model (see Supplementary Information).

Using the resulting stochastic time series model, we assess the expected probability distributions of record temperatures and, therefore, the likelihoods of individual record warm years and observed runs of record warmth. We evaluate the likelihood of specific temperature extremes given the particular realization of natural (volcanic and solar) forcing *N* that actually occurred. It is also possible to assess these extremes considering all possible realizations of natural variability (i.e. treating forced natural variability *N* as a random variable in addition to *I*), but this approach requires additional assumptions that are more tenuous (see Methods). We therefore choose to employ the first of these two approaches for the analyses presented here, though we demonstrate via separate analyses (See Supplementary Information) that the main findings are robust with respect to which approach is used.

Using the CMIP5 multi-model mean to estimate the total (anthropogenic + natural, forced long-term component (*F* = *A* + *N*) of temperature change (see Methods), we find good overall agreement with observations during the period (1880–2014) of overlap (Fig. 1). The simulated and observed warming in recent decades matches well, providing further context for recent discussions of putative inconsistencies between the two^11^,^12^,^13^,^14^,^15^,^16^,^17^,^18^,^19^,^20^,^21^ and lending additional support to the IPCC conclusion^22^ that there is little if any natural contribution to global warming over the past half century.

Of particular interest in the context of this study, however, is the residual term –the difference between the observations and simulations (Fig. 2)—as this forms the basis of our estimate of the internal natural variability component *I*, i.e. the “climate noise” (see Methods). In modeling *I*, we invoke several different alternative null hypotheses (see Methods). These include (1) AR(1) ‘red noise’ (the simplest defensible null hypothesis^23^) and (2) a more general linear stationary ARMA(p,q) noise process (which accommodates greater noise structure). Some researchers have argued that climate noise may exhibit first order non-stationary behavior, i.e. so-called “long-range dependence”. Analyses of both modern and paleoclimate observations, however, support the conclusion that it is only the anthropogenic climate change signal that displays first-order non-stationarity behavior, with climatic noise best described by a stationary noise model^24^. We have nonetheless considered the additional case of (3) ‘persistent’ red noise wherein the noise model is fit to the raw observational series. The latter model can be considered somewhat unphysical, as the inferred amplitude and persistence of the ‘noise’ are both inflated by the presence of the non-stationary anthropogenic-forced component. It is thus used as an extreme upper bound estimate on both the amplitude and persistence of climatic noise.

We generated a large ensemble (a million) surrogates for the internal variability *I* via Monte Carlo simulations using each of the three alternative noise models discussed above. The resulting internal variability surrogates were used (see Methods) to produce distributions of temperatures expected both with and without anthropogenic forcing. We tabulated the distributions obtained for individual years of interest (the ‘record’ years of 1998, 2005, 2010, and 2014) as well as ‘runs’ of record years including scenarios where 9 of the warmest 10 (“9/10 warmest”) and 13 of the warmest 15 years (“13/15 warmest”) occur since the year 2000. Our interest in the latter two statistics is motivated not by the fundamental significance of those particular statistics, but rather, by the *a priori* attention given them in recent media coverage of global warming. One could easily justify a variety of alternative statistics (and we have archived our Monte Carlo simulations so that others may indeed mine them for other statistical attributes).

In these analyses, we are asking whether or not these particular records are likely to have arisen in the absence of anthropogenic forcing but *with* the observed history of natural radiative forcing, so distinct years and intervals are unique in their statistical attributes. For example, 2010 falls near a local peak in negative radiative forcing by solar and volcanic activity (Supplementary Information), which makes record temperatures less likely at that time [In the alternative approach where natural radiatively-forced temperature change is itself treated as a random variable (see Methods and Supplementary Information), there is nothing special about any individual year or any particular 15 year interval].

Using the most defensible of the three noise null hypotheses (ARMA), we find (Fig. 3) that the recent run of record-breaking NH and global mean temperatures (13 of the 15 warmest years and 9 of the 10 warmest years having occurred since 2000) is extremely unlikely to have occurred due to natural variability alone, with odds of 1-in-170,000/1-in-5000 (NH) and 1-in-10,000/1-in-770 (global) respectively (Table 1). Qualitatively similar results are obtained regardless of (Supplementary Information) (a) which of the two approaches is used (anthropogenic-only or all-forcing experiments), (b) which surface temperature dataset (GISTEMP or HadCRUT4) is employed, (c) whether model surface air temperature is substituted for sea surface temperature over ocean regions, (d) whether or not simulations are restricted to only those that include both aerosol indirect effects and (e) whether a parametric or non-parametric Monte Carlo procedure is used. Regardless of which variant is employed, the odds for the 13/15 and 9/10 runs are no greater than 1-in-5000/1-in-670 (NH) and 1-in-1700/1-in-500 (global) respectively. While considerably greater than cited in some recent media reports, these odds are low enough to suggest that recent observed runs of record temperatures are extremely unlikely to have occurred in the absence of human-caused global warming.

We find it even less likely that natural variabillity might have produced the observed specific individual yearly temperature records. That is not especially surprising. Unlike the run statistics, these records require not only that a record occurred, but also that a particular threshold of record warmth was reached. We estimate odds (Table 1) of no more than 1-in-1,000,000 that the 2014 temperature record would have arisen from natural varability alone for both NH and the globe. That conclusion also holds for the other record years 1998, 2005, and 2010. Allowing for all of the potential variants in dataset and methodology (Supplementary Information), that conclusion remains true for 2014, but the odds are somewhat higher for 1998 in particular (as high as 1-in-50,000 for NH and 1-17,000 for globe).

If one instead employs the ‘persistent red noise’ null hypothesis (Fig. 4), the odds of breaking these records due to apparent natural variability are considerably greater. But even here, the odds are no better than 1-in-100 for the 13/15 run of record-breaking years, and no better than 1-in-80 for the 2014 temperature record, for either NH or globe (Table 1). In other words, even using a too-conservative null hypothesis of persistent red noise, the recent observed record warmth is still unlikely to have occurred from natural variability alone.

Finally, our analysis allows us to comment on the odds of the observed records having been observed, given that there *has been* anthropogenic warming (Fig. 5). We find that the 13/15 and 9/10 record temperature runs were very likely to have been observed with likelihoods of occurrence (Table 1) of 76% and 88% respectively (NH) and 72% and 83% respectively (globe). Accounting for all variants on the procedure and dataset (Supplementary Information), the likelihoods range from 41–84% and 68–92% respectively (NH) and 42–80% and 69–88% respectively (globe), indicating that these runs had at a minimum, nearly equal odds of having occured, regardless of the details of the analysis.

The 2014 record had a likelihood (Table 1) of 37% and 40% for the NH and globe respectively, i.e. a rather high likelihood of roughly one-in-three to one-in-two. Accounting for all variants (Supplementary Information), the likelihoods range from 16%–81% and 17%–80% for the NH and globe respectively. Interestingly, the 1998 record had a considerably lower likelihood of 17% and 15% for NH and globe respectively (Table 1) (and as low as ~3% and 4% respectively in certain variants). That observation is consistent with the notion that this record was somewhat of an outlier, having been substantially boosted by an unusually large (in fact, by some measures the largest) El Niño on record. The fact that it took an usually long time (7 years, until the 2005 global temperature record) for the 1998 record to be matched or exceeded is related, in our analysis, to the fact that it occurred earlier than expected given the trajectory of anthropogenic warming.

In summary, our results suggest that the recent record temperature years are are roughly 600 to 130,000 times more likely to have occurred under conditions of anthropogenic than in its absence. Our findings thus underscore the profound impact that anthropogenic forcing has already had on temperature extremes.

## Methods

### Estimation of Global and Hemispheric Mean Surface Temperatures

We analyze both global and Northern Hemisphere (NH) mean temperatures. These two quanitities are complimentary: the NH mean is better sampled observationally, but exhibits greater internal variability owing to the potential influence of changes in cross-equatorial heat transport^25^, a process that does not influence the global mean. While the GISTEMP observational surface temperature dataset arguably better deals with data gaps in the Arctic critical to capturing the dramatic high-latitude warming in recent decades, we perform parallel analyses (Supplementary Information) using the alternative HADCRUT4 dataset to establish the robustness of our results with respect to the choice of observational product.

Given that the observational NH and global mean surface temperature make use of a blend (‘TAS/TOS’) of Surface Air Temperature (‘TAS’) over land and Sea Surface Temperature (‘TOS’) over oceans, we computed hemispheric and global means similarly from the CMIP5 simulations. Recent work has demonstrated that an apparent divergence over the past decade between model-simulated and observed surface temperature seen in some past comparisons (e.g. the 2013 IPCC report^26^) are likely due at least in part to an improper “apples-and-oranges” comparison of model-simulated TAS with observed TAS/TOS^13^.

For completeness, we have nonetheless also performed parallel analyses wherein simulated TAS is used in place of blended TAS/TOS (Supplementary Information). We extend the CMIP5 series through 2014 using the estimates provided by ref. 13 (Supplementary Information).

### Modeling Temperature Distributions

Our approach for defining the mean structure (i.e. forced component) is similar to that employed by Mann *et al*. (2014)^11^ and Steinman *et al*. (2015)^12^ wherein an average is taken from a large number of simulations of climate models driven with estimated historical radiative forcing changes. Since the noise realization in each simulation is statistically independent, the multi-model ensemble average provides an estimate of the forced-only component of climate change as long as the number of simulations is suitably large. While Steinman *et al*.^12^ use linear regression to estimate the forced component from the multimodel mean, they find the regression coefficient to be indistinguishable from unity in the case of hemispheric means, so a simple multi-model mean can be used.

*F* is in this way estimated from the ensemble of CMIP5 anthropogenic + natural forced simulations (*N* = 44 models, *M* = 170 total simulations spanning 1861–2005—see Supplementary Information) analyzed by refs 11,12, while the anthropogenic-only response *A* in eq. 1 is estimated from the CMIP5 anthropogenic-only (See Supplementary Information) forced simulations (*N* = 8 models, *M* = 44 total simulations spanning 1861–2005). The natural-only response *N* in eq. 1 is then estimated as the difference *N* = *F-A*. We produce surrogates of the total natural variability *N* + *I* (total variability *F* + *I*) using the above estimate of *N* (*F*) and Monte Carlo surrogates for the internal variability *I* (see below).

We also considered an alternative approach (Supplementary Information) wherein the likelihood of extremes is assessed considering all possible realizations of the total natural variability (i.e. treating forced natural variability too as stochastic in nature). In that case, we estimate only the anthropogenic forced component (*A*) as mean structure. The remaining total natural (forced + internal) variability component *N* + *I* is estimated by subtracting the estimated mean structure (*A*) from the observations (*T*) to yield a residual that is again simulated with a stationary stochastic time series model. This choice is not entirely without justification since e.g. the timing and magnitude of volcanic eruptions might be viewed as a stochastic rather than deterministic process. The statistical characteristics of natural volcanic and solar forcing however differ from that of internal variability. Volcanic forcing is impulsive and one-sided (eruptions always cause cooling), and solar forcing has spectral peaks (associated with e.g. the ~11 year solar cycle), and the assumption that the resulting temperature variability can be represented by a stationary time series model can be questioned. We address this concern testing the adequacy of the resulting statistical model, which yields some additional caveats (see Supplementary Information).

### Null Hypotheses Tested

The natural variability component of the temperature time series can be modeled under a variety of null hypotheses, the simplest of which is the (i) first order autoregressive i.e. AR(1) ‘red noise’ process. For such a process, the effective degrees in tests of the mean are *N’* = [(1 − ρ)/(1 + ρ)] *N* where *N* is the number of years of data, and 0 ≤ ρ ≤ 1 is the lag-one autocorrelation coefficient of the annual data series. For ρ = 0 we recover the result for an uncorrelated series *N’* = *N*, while as ρ → 1, the effective degrees of freedom in the sample approaches *N’* = 0 regardless of the size of the sample.

Such a statistical model may not adequately account for additional sources of natural variability, e.g. oscillatory temperature variations due to phenomena such as El Niño. We thus alternatively employ (ii) the more general linear auto-regressive/moving average ARMA(*p*,*q*) process, which accomodates considerably more statistical structure, to characterize the natural variablity (noise) in the the temperature series. The orders *p*,*q* are selected by the conservative Bayesian Information Criterion (BIC). Note that in some cases (i.e. when *p* = 1, *q* = 0 is selected), the chosen ARMA model reduces to AR(1). Evaluation of the resulting statistical models including statistical significance of model parameters and tests of model adequacy are provided in the Supplementary Information.

Finally, we consider (iii) an overly conservative model of ‘persistent red noise’ for the natural variability. This model again takes the form of an AR(1) process, but the autocorrelation ρ is instead now estimated not from the derived natural variability component but from the raw temperature series itself. This models yields higher levels of apparent “persistence” (i.e. considerably larger low-frequency fluctuations) because long-term trends associated with anthropogenic forcing are instead now treated as if they reflect natural low-frequency fluctuations. As this artificially inflates the apparent amplitude of long-term natural variability, the model can be argued to provide an upper bound on the noise amplitude and persistence.

The noise parameters were diagnosed from the residuals over the entire (1880–2014) period of overlap between model-simulated and observed temperatures. For completeness, however, we performed additional experiments (Supplementary Information) wherein noise parameters are estimated only from the sub-intervals of (i) 1880–2005 and (ii) 1880–1999. These experiments demonstrate that (i) our findings are not meaningfully influenced by the post-2005 extrapolation of the CMIP5-simulated temperatures and (ii) our inferences regarding record temperatures during the interval 2000–2014 are robust to whether or not that time interval is included in the fitting of the noise model. In fact, the chance likelihoods of the observed warmth being due to natural variability under the ‘persistent red noise’ assumption are even lower in these cases (Supplementary Information), as the estimated noise amplitude and persistence are further inflated by ongoing anthropogenic warming under the persistent noise assumption, so the use of the more recent data (i.e. through 2014) increases the likelihoods of chance occurrence.

## Additional Information

**How to cite this article**: Mann, M. E. *et al*. The Likelihood of Recent Record Warmth. *Sci. Rep.* **6**, 19831; doi: 10.1038/srep19831 (2016).

## Supplementary Material

Supplementary Information

## Figures and Tables

**Figure 1:**
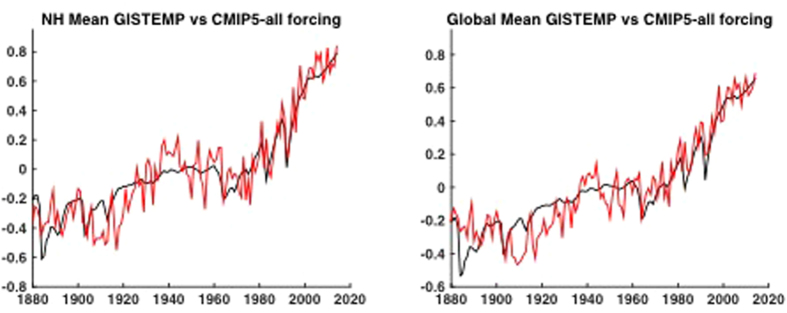
NH (left) and Global (right) mean temperature observations (GISTEMP-red) vs. CMIP5 mean estimated forced component (black) using CMIP5 all-forcing experiments. Here and in subsequent figures, model hemispheric and global mean surface temperatures are based on a combination (“TAS/TOS”) of surface air temperature (“TAS”) over land and sea surface temperature (“TOS”) over ocean. Here and elsewhere in the article, temperatures are defined as anomalies relative to the the long-term (1880-2014) mean.

**Figure 2:**
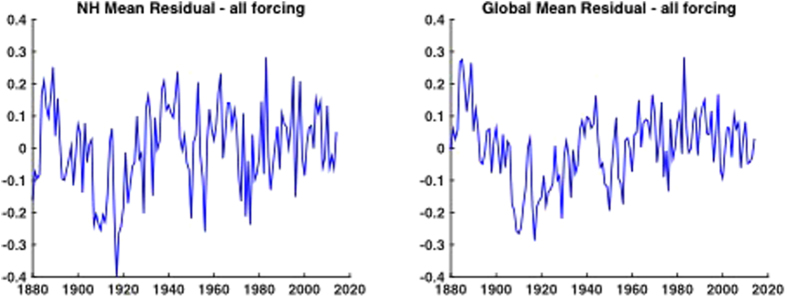
NH (left) and Global (right) mean residual components after CMIP5-estimated forced component is subtracted from (GISTEMP) observational temperatures using CMIP5 all- forcing experiments (AD 1880–2014).

**Figure 3:**
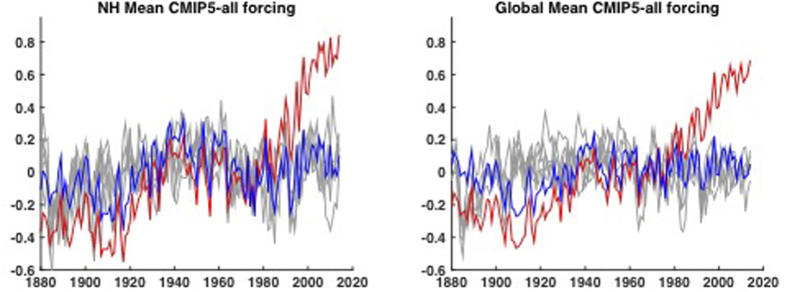
NH (left) and Global (right) mean estimated natural variability component (blue) along with five different Monte Carlo ARMA surrogates (gray) using CMIP5 all-forcing experiments. Shown for comparison are the raw observational series (red) (AD 1880–2014).

**Figure 4:**
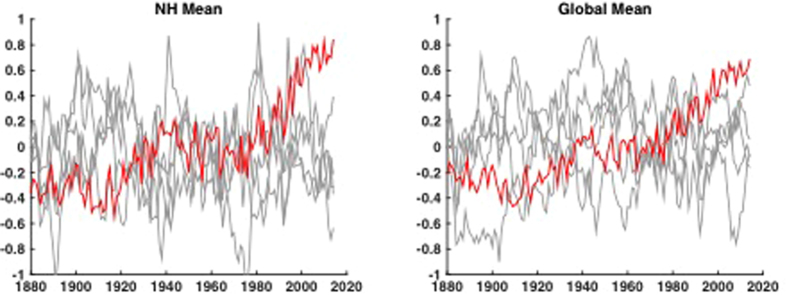
NH (left) and Global (right) mean temperature natural variability component associated with five different Monte Carlo Persistent Red Noise realizations (gray). Shown for comparison are the raw observational series (red) (AD 1880–2014).

**Figure 5:**
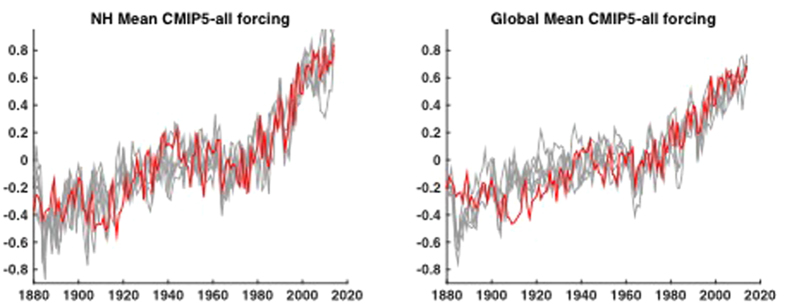
NH (left) and Global (right) mean temperature series along with five different Monte Carlo surrogates based on forced signal + ARMA noise realizations (as shown in Fig. 2, gray) using CMIP5 all-forcing experiments. Shown for comparison are the raw observational series (red) (AD 1880–2014).

**Table 1:** Estimated Likelihoods (in %).

Experiment – All Forcings	1998	2005	2010	2014	9/10	13/15
NH GISTEMP TAS/TOS AR(1)	10^−4^	<10^−4^	<10^−4^	<10^−4^	9·10^−4^	<10^−4^
NH GISTEMP TAS/TOS ARMA(1,1)	<10^−4^	<10^−4^	<10^−4^	<10^−4^	0.02	6·10^−4^
NH GISTEMP TAS/TOS ARMA(1,1) w/ Anthro	6.0	13	16	37	88	76
Glb GISTEMP TAS/TOS AR(1)	<10^−4^	<10^−4^	<10^−4^	<10^−4^	0.01	5·10^−4^
Glb GISTEMP TAS/TOS ARMA(1,1)	<10^−4^	<10^−4^	<10^−4^	<10^−4^	0.13	0.01
Glb GISTEMP TAS/TOS ARMA(1,1) w/ Anthro	6.8	18	23	40	83	72
Experiment – Persistent Red Noise	1998	2005	2010	2014	9/10	13/15
NH GISTEMP Persistent Red Noise	2.9	1.8	1.0	1.1	1.7	0.5
Glb GISTEMP Persistent Red Noise	2.9	2.1	1.4	1.2	2.5	1.0
