# Supplementary material for: The Likelihood of Recent Record Warmth
Source: Sci Rep. 2016 Jan 25;6:19831. doi: 10.1038/srep19831 (PMC4726353; doi:10.1038/srep19831)
Supplement: Supplementary Information [file srep19831-s1.doc]

# The Likelihood of Recent Record Warmth

**Michael E. Mann*^1^, Stefan Rahmstorf^2^, Byron A. Steinman^3^, Martin Tingley^4^, Sonya K. Miller^1^**^1^Department of Meteorology, Pennsylvania State University ^2^Earth System Analysis, Potsdam Institute for Climate Impact Research ^3^Large Lakes Observatory and Department of Earth and Environmental Sciences, University of Minnesota-Duluth ^4^Departments of Meteorology and Statistics, Pennsylvania State University

**Supplementary Information**

**Further Details**

***Supplementary Results***

Counterparts to Figures 1-3 and 5 of the main article are shown for the anthropogenic-only forcing experiment in Figures S1-S4 respectively. Counterparts to Figures 1-3 of the main article are also shown for the all-forcing case where (a) Model TAS is substituted for TAS/TOS blend (Figure S5), (b) HadCRUT4 substituted for GISTEMP in the analysis (Figure S6) and (c), Model AIE simulations only are used (Figure S7). Details about the CMIP5 models used in both the all-forcing and anthropogenic-only forcing experiments are provided in Table S1.

***Updating CMIP5 Series through 2014:***

For the anthropogenic-only experiments, we smoothed the NH and global CMIP5 multimodel mean series on a multidecadal time scale (filter retaining 40 year and longer-term variabilty) to remove the small residual interannual variability that results from the finite size of the ensemble (Figure S8). The resulting series are remarkably linear over the past several decades, motivating a simple linear extension beyond the 2005 termination date to 2014 (we extrapolate the linear trend over the 20 year 1986-2005 period to the 2014 boundary). This is essentially equivalent to using a business-as-usual (“BAU”) 21^st^ century RCP scenario to extend the series, as is often done. Such a procedure however, neglects documented changes in anthropogenic radiative forcing over the past decade (ref 12 of main article). We thus incorporate the ref. 12 corrected anthropogenic forcing estimates (these provide corrected anthropogenic forcing from 2006-2013, which we extend to 2014 by persistence of the 2013 value; the estimates also include to the CMIP5 multimodel mean forced response back to 1986). For the CMIP5 all-forcing (i.e. anthropogenic+natural forcing) multimodel mean, we make use of the ref 12. corrections to both the anthropogenic and natural radiatively forced response.

***Estimating the natural forcing-only CMIP5 multimodel mean:***

A “natural-only” forced CMIP5 multimodel series is obtained simply by differencing the anthropogenic-only and all-forcing CMIP5 mulitmodel mean series. (Figure S9).

***Details of Statistical Modeling Exercises:***

The ARMA(*p*,*q*) model contains *p* autoregressive terms (the “AR” part of the model) and *q* moving-average terms (the “MA” part of the model), taking the form:

*y_t_* = c + [*a_1_ y_t-1_* + … + *a_p_* *ε_t-p_* ] + [*b_1_ ε_t-1_* + … + *b_q_ ε_t-p_* ] + *ε_t_*

where the “innovation” sequence *ε_t_* is assumed to conform to Gaussian white noise. The AR(1) “red noise” model is a special simplified case.

The selection of *p* and *q* in the ARMA(*p*,*q*) time series model for each series was accomplished by minimizing the Bayesian Information Criterion (BIC) among all values of *p* and *q* tested (up through a suitably chosen upper limit of *p=q=*10) which is calculated based on the log likelihood function and number of parameters *n=p+q+1* for each fitted model.

*Standard Case: modeling internal variability (I in eq. 1 of main article):*

Statistical model parameter values, standard errors, and associated t statistics for NH and global mean temperature for the standard case (“all forcing” experiments) featured in the main article are provided in Table S2 (top). Values are given for each of the statistical model parameters of the ARMA(p,q) selected model. We see that each of the model parameters of each selected model is highly significant (the smallest t statistic for either of the parameters for either of the series modeled is t=3.07, which is significant at the *p*=0.002 level for a two-sided test with *N*=135).

Equally important in establishing the reliability of the selected statistical models are tests of model adequacy, namely establishing that the estimated innovation sequence is consistent with white noise Gaussian behavior, as assumed by the statistical modeling exercise. In Figure S10 (top), we show the autocorrelation of the innovation sequence out to lag 20 for each of the two series modeled. There is no evidence of any structure that is inconsistent with the assumption of Gaussian white noise (i.e. where the value of the autocorrelation function exceeds the 95% two-sided statistical significance limits).

*Alternative Case: modeling total nature variability (N+I in eq. 1 of main article):*

Statistical model parameter values, standard errors, and associated t statistics for NH and global mean temperature are also provided for the alternative case (“anthropogenic-only forcing” experiments) in Table S2 (bottom). In this case too, each of the model parameters of each selected model is highly significant.

In this case, however, there are some caveats with respect to the issue of model adequacy when we look at the autocorrelation of the innovation sequence (Figure S10, bottom). For one of the two series (global mean) there is evidence of structure that is (modestly) inconsistent with the assumption of Gaussian white noise (i.e. where the value of the autocorrelation function exceeds the 95% two-sided statistical significance limits).

Additional caveats thus apply for that experiment. We speculate that the failure in this case for the innovation sequence to satisfy the requirements of Gaussian white noise behavior arises from the non-Gaussian nature of natural external forcing events (e.g. the impulse-like cooling associated with volcanic forcing). As discussed in the main article, this behavior would appear to present a limitation in modeling forced natural variability using a stationary time series model. This limitation should also apply to the NH mean anthropogenic-only forcing experiment, yet there is no evidence of non-random structure in the innovation sequence in that case. We suspect that is because of the greater relative important of internal variability in the NH mean relative to the global mean. Natural radiatively-forced temperature changes as a result account for a larger share of the total natural variability in global mean temperature, and so the deficiency is more readily apparent in the characteristics of the innovation sequence.

***Monte Carlo Simulation Results***

Statistical model parameter values, standard errors, and associated t statistics for NH and global mean temperature in both the “all forcing” experiments featured in the main article and the alternative “anthropogenic-only “ forcing experiments, are provided in Table S2. Values are given for each of the statistical model parameters of the ARMA(p,q) model selected by BIC (see Methods in main article). We see that each of the model parameters of each selected model is highly significant (the smallest t statistic for any of the parameters in any of the four cases is t=3.07, which is significant at the *p*=0.002 level for a two-sided test with *N*=135).

Using the ARMA(1,1) noise model favored by BIC and the scenario wherein forced natural temperature variation is specified *a priori* (i.e. the all-forcing case) we estimate (Table 1 of main article) for the NH mean temperature a likelihood of 6·10^-4^ % for 13/15 warmest, i.e. odds of roughly 1-in-170,000 in the absence of anthropogenic warming. We obtain a considerably greater likelihood of 0.02% (1-in-5000) for 9/10 warmest. While 9/10 might initially seem less likely than 13/15 to occur by chance, the opposite is actually the case, given the underlying combinatorics of considering 13 vs. 9 years. When forced natural variability is treated instead as a random variable (i.e. the anthropogenic-only forcing case—see Table S3), we obtain considerably higher likelihoods for chance occurance for both 13/15 (0.01%, i.e. odds of roughly one-in-10,000) and 9/10 (0.1%, i.e. odds of roughly 1-in-1000). The recent negative natural radiative forcing contribution makes recent record temperature runs considerably less likely to have occurred by chance when that forcing history is taken into account. Use of the AR(1) model gives lower probabilities of chance occurance of these runs than the more structured ARMA model.

The record NH temperatures of 2005, 2010, 2014 each have a likelihood of <10^-4^ % (odds of less than one-in-a-million) of having occurred in the absence of anthropogenic global warming. The slightly cooler 1998 record has a higher likelihood of 6·10^-4^ % (odds of one-in-170,000) according to the anthropogenic-only experiments. For global mean temperature, the favoured ARMA(1,1) model yields, for the all-forcing experiments, likelihoods of 0.01% (1-in-10,000) for 13/15 warmest and 0.13% (roughly 1-in-800) for 9/10 warmest, with record temperatures in 1998, 2005, 2010, 2014 each having a a likelihood of <10^-4^ % (odds of less than 1-in-1,000,000).

For the model of persistent red noise, we unsuprisingly find substantially greater odds of observing record temperatures naturally, but even here those odds are rather low. We estimate for the NH mean temperature (Table 1 of main article) a likelihood of 0.5% (1-in-200) for 13/15 warmest and 1.7% (roughly 1-in-60) for 9/10 warmest, in the absence of anthropogenic warming. The individual record years of 2005, 2010, 2014 each have a likelihood of between 1.1% and 1.8% (odds between 1-in-50 and 1-in-100), while the 1998 temperature record has a slightly greater likelihood of 2.9% (roughly 1-in-30). For global mean temperature, we obtain similar likelihoods of 1.0% (1-in-100) for 13/15 warmest and 2.5% (1-in-40) for 9/10 warmest, while 2005, 2010, 2014 record years have likelihoods between 1.2 and 2.1% (odds between 1-in-50 and 1-in-80), with 1998 again a slightly greater likelihood of 2.9% (1-in-30).

When we actually account for anthropogenic warming by adding the CMIP5 anthropogenic temperature signal to the natural variability series, we observe high degrees of likelihood for having observed the recent record temperatures. We estimate for the NH mean temperature (Table 1 of main article) likelihoods for 13/15 warmest of ~48% and 76% (roughly 1-in-2 and 3-in-4) and likelihoods for 9/10 warmest of ~73% and 88% (roughly 3-in-4 and 9-in-10) for anthropogenic-only and all-forcing experiments respectively. Results for global mean temperature are very similar to those for NH mean temperature. The fact that recent record temperatures are consistently more likely to have occurred in the all-forcing scenario arises from the net positive long-term trend in natural radiative forcing (due primarily to the large negative forcing during the late 19^th^ century--see Figure S9), which leads to warmer predicted recent temperatures in the all-forcing case (compare lower and upper panels in Figure 1 of main article). The individual record years of 2005, 2010, and 2014 have likelihoods of 8-40%, depending on whether NH or global mean temperatures are used, and whether the all-forcing or anthropogenic-only experiments are used. The 1998 temperature record has a substantially lower likelihood of 2-7%.

Results are qualitatively similar to those described above if (a) model TAS is used in place of TAS/TOS (Table S4), (b) HadCRUT4 is used in place of GISTEMP (Table S5), (b) a non-parameteric bootstrap is used in the Monte Carlo procedure in place of Gaussian innovations (Table S6), (c) simulations are restricted to only those models (see Table S1) that include both 1^st^ and 2nd aerosol indirect effects ( “AIE”— Table S7) (note that this analysis was not possible for the anthropogenic-only simulations, in which case only *N*=2 models/*M*=6 total realizations are available), and (d) statistical parameters are estimated based on data through either 1999 or 2005 (rather than through 2014 as in all other experiments) (Table S8). There are some quantitative differences that are however noteworthy. For the AIE experiments, the likelihood of the 1998 global temperature record from natural variability alone rises to 0.006% (1-in-170,000), while the likelihood of the 9/10 record streak climbs to 0.2% (1-in-500). When HadCRUT4 is used in place of GISTEMP, the persistent red noise experiments yield a likeilhood of nearly 4% for the 1998 record arising from natural variability. When SAT is used in place of SST/SAT and global warming is accounted for, the likelihood of the 1998 NH temperature records exceeds 20% (1-in-5), the likelihood of the 2014 record exceeds 80% (4-in-5) and the likelihood of 9/10 record streak exceeds 90% (9-in-10). When statistical parameters are estimated based on data through either 1999 or 2005, the likelihoods are lower for the persistent noise simulations. This occurs because the noise amplitude and persistence are further inflated by the ongoing anthropogenic warming through 2014 in this case, so the use of the more recent data (i.e. through 2014) increases the likelihoods of chance occurrence.

As a general rule, higher likelihoods of chance occurrence result from using model mean SAT, employing AIE simulations only or the anthropogenic-only experiments, owing to the larger systematic differences between model and observations (and hence, the apparent natural variability). In the case where model mean TAS is used, the CMIP5 models warm too much relative to observations in recent decades (Figure S5) while considering AIE simulations only, the model means warm too little (Figure S7).

**Supplementary Tables and Figures**

**Table S1**. CMIP5 Climate Model Simulations

| Model | Number of Realizations | Length of historical runs (yr) | Start year AD | End Year AD | 1^st^ *and* 2^nd^ aerosol indirect effects |
| --- | --- | --- | --- | --- | --- |
| *All Forcing Simulations* | | | | | |
| GISS-E2-R | 24 | 156 | 1850 | 2005 | N |
| GISS-E2-H | 17 | 156 | 1850 | 2005 | N |
| CNRM-CM5 | 10 | 156 | 1850 | 2005 | N |
| CSIRO-Mk3.6.0 | 10 | 156 | 1850 | 2005 | Y |
| GFDL-CM2.1 | 10 | 145 | 1861 | 2005 | N |
| HadCM3 | 10 | 146 | 1860 | 2005 | N |
| CCSM4 | 6 | 156 | 1850 | 2005 | N |
| IPSL-CM5A-LR | 6 | 156 | 1850 | 2005 | N |
| CanESM2 | 5 | 156 | 1850 | 2005 | N |
| GFDL-CM3* | 5 | 146 | 1860 | 2005 | Y |
| HadGEM2-ES | 5 | 146 | 1860 | 2005 | Y |
| MIROC5 | 5 | 163 | 1850 | 2012 | Y |
| MRI-CGCM3 | 4 | 156 | 1850 | 2005 | Y |
| ACCESS1.3 | 3 | 156 | 1850 | 2005 | Y |
| bcc-csm1-1 | 3 | 163 | 1850 | 2012 | N |
| bcc-csm1-1m | 3 | 163 | 1850 | 2012 | N |
| CESM1-CAM5 | 3 | 156 | 1850 | 2005 | Y |
| CESM1-FASTCHEM | 3 | 156 | 1850 | 2005 | N |
| FIO-ESM | 3 | 156 | 1850 | 2005 | N |
| IPSL-CM5A-MR | 3 | 156 | 1850 | 2005 | N |
| MPI-ESM-MR** | 3 | 156 | 1850 | 2005 | N |
| MIROC-ESM | 3 | 156 | 1850 | 2005 | Y |
| MPI-ESM-LR* | 3 | 156 | 1850 | 2005 | N |
| NorESM1-M | 3 | 156 | 1850 | 2005 | Y |
| MPI-ESM-P** | 2 | 156 | 1850 | 2005 | N |
| CESM1-WACCM | 1 | 156 | 1850 | 2005 | N |
| HadGEM2-CC | 1 | 146 | 1860 | 2005 | Y |
| HadGEM2-AO** | 1 | 146 | 1860 | 2005 | Y |
| ACCESS1.0 | 1 | 156 | 1850 | 2005 | Y |
| BNU-ESM | 1 | 156 | 1850 | 2005 | N |
| CESM1-BGC | 1 | 156 | 1850 | 2005 | N |
| CMCC-CESM | 1 | 156 | 1850 | 2005 | N |
| CMCC-CM | 1 | 156 | 1850 | 2005 | N |
| CMCC-CMS | 1 | 156 | 1850 | 2005 | N |
| CNRM-CM5-2 | 1 | 156 | 1850 | 2005 | N |
| GFDL-ESM2G | 1 | 145 | 1861 | 2005 | N |
| GFDL-ESM2M | 1 | 145 | 1861 | 2005 | N |
| GISS-E2-H-CC | 1 | 161 | 1850 | 2010 | N |
| GISS-E2-R-CC | 1 | 161 | 1850 | 2010 | N |
| INM-CM4 | 1 | 156 | 1850 | 2005 | N |
| IPSL-CM5B-LR | 1 | 156 | 1850 | 2005 | N |
| MRI-ESM1 | 1 | 155 | 1851 | 2005 | Y |
| FGOALS-g2** | 1 | 156 | 1850 | 2005 | Y |
| NorESM1-ME | 1 | 156 | 1850 | 2005 | Y |
| *Anthropogenic Simulations* | | | | | |
| CNRM-CM5 | 10 | 163 | 1850 | 2012 | N |
| GISS-E2-H | 10 | 163 | 1850 | 2012 | N |
| GISS-E2-R | 10 | 163 | 1850 | 2012 | N |
| CCSM4 | 4 | 156 | 1850 | 2005 | N |
| CESM1-CAM5 | 3 | 156 | 1850 | 2005 | Y |
| GFDL-CM3 | 3 | 146 | 1860 | 2005 | Y |
| IPSL-CM5A-LR | 3 | 156 | 1850 | 2005 | N |
| GFDL-ESM2M | 1 | 145 | 1861 | 2005 | N |
| *One realization from this model was not included in the SAT/SST model means. | | | | | |
| ** This model was not included in the SAT/SST model means.  MOdel  Model in bold | | | | | |
|  | | | | | |

**Table S2**. Estimated Likelihoods (in %) – Anthropogenic-Only Forcing Experiments

| Experiment – All Forcings – NH – ARMA(1,1) | value | Standard error | t statistic |  | |
| --- | --- | --- | --- | --- | --- |
| AR(1) coefficient | 0.7875 | 0.1142 | 6.90 |  | |
| MA(1) coefficent | -0.4710 | 0.1535 | -3.07 |  | |
|  |  |  |  | | |
| Experiment – All Forcings- Globe – ARMA(1,1) | 1998 | 2005 | 13/15 | |  |
| AR(1) coefficient | 0.8587 | 0.07438 | 11.6 | |  |
| MA(1) coefficent | -0.4472 | 0.1172 | -3.81 | |  |

| Experiment – Anthro only – NH - ARMA(1,1) | value | Standard error | t statistic |  | |
| --- | --- | --- | --- | --- | --- |
| AR(1) coefficient | 0.9188 | 0.05067 | 18.13 |  | |
| MA(1) coefficent | -0.6240 | 0.1020 | -6.11 |  | |
|  |  |  |  | | |
| Experiment – Anthro only - Globe – AR(1) | 1998 | 2005 | 13/15 | |  |
| AR(1) coefficient | 0.586 | 0.06720 | 8.72 | |  |

**Table S3**. Estimated Likelihoods (in %) – Anthropogenic-Only Forcing Experiments

|  |  |  |  |  | | |  |
| --- | --- | --- | --- | --- | --- | --- | --- |
| Experiment – Anthropogenic Forcing Only | 1998 | 2005 | 2010 | 2014 | 9/10 | 13/15 |  |
| NH GISTEMP TAS/TOS AR(1) | 10^-4^ | <10^-4^ | <10^-4^ | <10^-4^ | 0.005 | <10^-4^ |  |
| NH GISTEMP TAS/TOS ARMA(1,1) | 6·10^-4^ | 10^-4^ | <10^-4^ | 10^-4^ | 0.1 | 0.01 |  |
| NH GISTEMP TAS/TOS ARMA(1,1) w/ Anthro | 5.2 | 9.3 | 16 | 28 | 73 | 48 |  |
| Glb GISTEMP TAS/TOS AR(1)/ARMA(1,0) | <10^-4^ | <10^-4^ | <10^-4^ | <10^-4^ | 0.006 | <10^-4^ |  |
| Glb GISTEMP TAS/TOS ARMA(1,0) w/ Anthro | 2.0 | 8.3 | 21 | 34 | 75 | 50 |  |

**Table S4**. Estimated Likelihoods (in %) – Model SAT in place of SAT/SST

| Experiment – All Forcings | 1998 | 2005 | 2010 | 2014 | 9/10 | 13/15 |  |
| --- | --- | --- | --- | --- | --- | --- | --- |
| NH GISTEMP TAS AR(1) | 10^-4^ | <10^-4^ | <10^-4^ | <10^-4^ 0.001 10^-4^ | 0.001 10^-4^ | 10^-4^ |  |
| NH GISTEMP TAS ARMA(1,1) | <10^-4^ | <10^-4^ | <10^-4^ | <10^-4^ | 0.02 | 7·10^-4^ |  |
| NH GISTEMP TAS ARMA(1,1) w/ Anthro | 20 | 43 | 55 | 81 | 92 | 84 |  |
| Glb GISTEMP TAS AR(1) | <10^-4^ | <10^-4^ | <10^-4^ | <10^-4^ | 0.01 | 5·10^-4^ |  |
| Glb GISTEMP TAS ARMA(1,1) | <10^-4^ | <10^-4^ | <10^-4^ | <10^-4^ | 0.15 | 0.02 |  |
| Glb GISTEMP TAS ARMA(1,1) w/ Anthro | 21 | 47 | 60 | 80 | 88 | 80 |  |
|  |  |  |  |  | | |  |
| Experiment – Anthropogenic Forcing Only | 1998 | 2005 | 2010 | 2014 | 9/10 | 13/15 |  |
| NH GISTEMP TAS AR(1) | <10^-4^ | <10^-4^ | <10^-4^ | <10^-4^ | 0.005 | 10^-4^ |  |
| NH GISTEMP TAS ARMA(1,1) | 2·10^-4^ | <10^-4^ | <10^-4^ | <10^-4^ | 0.13 | 0.01 |  |
| NH GISTEMP TAS ARMA(1,0) w/ Anthro | 15 | 29 | 45 | 65 | 83 | 61 |  |
| Glb GISTEMP TAS AR(1)/ARMA(1,0) | 0.001 | <10^-4^ | <10^-4^ | <10^-4^ | 0.003 | 10^-4^ |  |
| Glb GISTEMP TAS ARMA(1,0) w/ Anthro | 11 | 33 | 60 | 78 | 84 | 61 |  |

**Table S5**. Estimated Likelihoods (in %) – HadCRUT4 in place of GISTEMP

| Experiment – All Forcings | 1998 | 2005 | 2010 | 2014 | 9/10 | 13/15 |  |
| --- | --- | --- | --- | --- | --- | --- | --- |
| NH HadCRUT4 TAS/TOS AR(1) /ARMA(1,0) | <10^-4^ | <10^-4^ | <10^-4^ | <10^-4^ | 7·10^-4^ | <10^-4^ |  |
| NH HadCRUT4 TAS/TOS ARMA(1,0) w/ Anthro | 14 | 18 | 31 | 59 | 89 | 76 |  |
| Glb HadCRUT4 TAS/TOS AR(1)/ARMA(1,0) | 10^-4^ | 1·10^-4^ | <10^-4^ | <10^-4^ | 0.001 | <10^-4^ |  |
| Glb HadCRUT4 TAS/TOS ARMA(1,0) w/ Anthro | 7.2 | 28 | 35 | 59 | 75 | 50 |  |
|  |  |  |  |  | | |  |
| Experiment – Anthropogenic Forcing Only | 1998 | 2005 | 2010 | 2014 | 9/10 | 13/15 |  |
| NH HadCRUT4 TAS/TOS AR(1) /ARMA(1,0) | 0.001 | <10^-4^ | <10^-4^ | <10^-4^ | 0.005 | 10^-4^ |  |
| NH HadCRUT4 TAS/TOS ARMA(1,1) w/ Anthro | 9.9 | 11 | 26 | 44 | 68 | 41 |  |
| Glb HadCRUT4 TAS/TOS AR(1)/ARMA(1,0) | <10^-4^ | <10^-4^ | <10^-4^ | <10^-4^ | 0.005 | <10^-4^ |  |
| Glb HadCRUT4 TAS/TOS ARMA(1,0) w/ Anthro | 4.0 | 18 | 34 | 51 | 69 | 42 |  |
|  |  |  |  |  | | |  |
| Experiment – Persistent Red Noise | 1998 | 2005 | 2010 | 2014 | 9/10 | 13/15 |  |
| NH HadCRUT4 Persistent Red Noise | 3.5 | 1.6 | 1.2 | 1.4 | 1.6 | 0.5 |  |
| Glb HadCRUT4 Persistent Red Noise | 2.8 | 2.5 | 1.6 | 1.6 | 1.9 | 0.7 |  |

**Table S6**. Estimated Likelihoods (in %) – bootstrap in place of Gaussian resampling

| Experiment – All Forcings | 1998 | 2005 | 2010 | 2014 | 9/10 | 13/15 |
| --- | --- | --- | --- | --- | --- | --- |
| NH GISTEMP TAS/TOS AR(1) | <10^-4^ | <10^-4^ | <10^-4^ | <10^-4^ | 0.002 | <10^-4^ |
| Glb GISTEMP TAS/TOS AR(1) | 10^-4^ | 10^-4^ | <10^-4^ | <10^-4^ | 0.01 | 6·10^-4^ |
|  |  |  |  |  | | |
| Experiment – Anthropogenic Forcing Only | 1998 | 2005 | 2010 | 2014 | 9/10 | 13/15 |
| NH GISTEMP TAS/TOS AR(1) | <10^-4^ | <10^-4^ | <10^-4^ | <10^-4^ | 0.005 | 10^-4^ |
| Glb GISTEMP TAS/TOS AR(1) | <10^-4^ | <10^-4^ | <10^-4^ | <10^-4^ | 0.01 | <10^-4^ |
|  |  |  |  |  | | |
| Experiment – Persistent Red Noise | 1998 | 2005 | 2010 | 2014 | 9/10 | 13/15 |
| NH GISTEMP TAS/TOS AR(1) | 2.0 | 1.0 | 0.7 | 0.7 | 1.3 | 0.3 |
| Glb GISTEMP TAS/TOS AR(1) | 3.4 | 2.3 | 1.4 | <1.2 | 2.6 | 1.1 |

**Table S7**. Estimated Likelihoods (in %) – A1E Simulations Only

| Experiment – All Forcings | 1998 | 2005 | 2010 | 2014 | 9/10 | 13/15 |
| --- | --- | --- | --- | --- | --- | --- |
| NH GISTEMP TAS/TOS AR(1) | 0.002 | 4·10^-4^ | <10^-4^ | <10^-4^ | 0.003 | 10^-4^ |
| NH GISTEMP TAS/TOS ARMA(1,1) | 0.002 | 9·10^-4^ | 2·10^-4^ | <10^-4^ | 0.15 | 0.02 |
| NH GISTEMP TAS/TOS ARMA(1,1) w/ Anthro | 2.6 | 6.6 | 7.0 | 16 | 78 | 60 |
| Glb GISTEMP TAS/TOS AR(1) | 9·10^-4^ | 3·10^-4^ | 10^-4^ | <10^-4^ | 0.01 | 7·10^-4^ |
| Glb GISTEMP TAS/TOS ARMA(1,1) | 0.006 | 0.003 | <10^-4^ | <10^-4^ | 0.2 | 0.06 |
| Glb GISTEMP TAS/TOS ARMA(1,1) w/ Anthro | 4.1 | 11 | 11 | 17 | 70 | 56 |

**Table S8**. Estimated Likelihoods (in %) – Data through 2005/1999 used for parameters

| Experiment – All Forcings – 2005 | 1998 | 2005 | 2010 | 2014 | 9/10 | 13/15 |
| --- | --- | --- | --- | --- | --- | --- |
| NH GISTEMP TAS/TOS AR(1) | <10^-4^ | <10^-4^ | <10^-4^ | <10^-4^ | 1·10^-3^ | <10^-4^ |
| NH GISTEMP TAS/TOS ARMA(1,1) | 2·10^-4^ | <10^-4^ | <10^-4^ | <10^-4^ | 0.02 | 8·10^-4^ |
| NH GISTEMP TAS/TOS ARMA(1,1) w/ Anthro | 6.4 | 13 | 17 | 37 | 87 | 74 |
|  |  |  |  |  | | |
| Experiment – Persistent Red Noise – 2005 | 1998 | 2005 | 2010 | 2014 | 9/10 | 13/15 |
| NH GISTEMP Persistent Red Noise | 1.3 | 0.7 | 0.4 | 0.4 | 0.9 | 0.2 |
|  |  |  |  |  | | |
| Experiment – All Forcings – 1999 | 1998 | 2005 | 2010 | 2014 | 9/10 | 13/15 |
| NH GISTEMP TAS/TOS AR(1) | 2·10^-4^ | <10^-4^ | <10^-4^ | <10^-4^ | 2·10^-3^ | <10^-4^ |
| NH GISTEMP TAS/TOS ARMA(1,1) | <10^-4^ | <10^-4^ | <10^-4^ | <10^-4^ | 0.03 | 0.001 |
| NH GISTEMP TAS/TOS ARMA(1,1) w/ Anthro | 6.3 | 13 | 17 | 37 | 86 | 73 |
|  |  |  |  |  | | |
| Experiment – Persistent Red Noise – 1999 | 1998 | 2005 | 2010 | 2014 | 9/10 | 13/15 |
| NH GISTEMP Persistent Red Noise | 0.5 | 0.2 | 0.1 | 0.1 | 0.5 | 0.1 |
|  |  |  |  |  | | |

**Figure S1.** NH (left) and Global (right) mean temperature observations (GISTEMP-red) vs. CMIP5 mean estimated forced component (black) using CMIP5 anthropogenic-only forcing experiments (AD 1880-2014).

**Figure S2.** NH (left) and Global (right) mean residual components after CMIP5-estimated forced component is subtracted from (GISTEMP) observational temperatures using CMIP5 anthropogenic-only forcing experiments (AD 1880-2014).

**Figure S3.** NH (left) and Global (right) mean temperature natural variability component associated with five different Monte Carlo Persistent Red Noise realizations (gray) using CMIP5 anthropogenic-only experiments. Shown for comparison are the raw observational series (red) (AD 1880-2014).

**Figure S4.** NH (left) and Global (right) mean temperature natural variability component associated with five different Monte Carlo Persistent Red Noise realizations (gray) using CMIP5 anthropogenic-only experimenmts. Shown for comparison are the raw observational series (red) (AD 1880-2014).

**Figure S5.** As in Fig. 1 (top), Fig. 3 (middle), and Fig. 5 (bottom) of main article, but using model TAS in place of model TAS/TOS in the analysis.

**Figure S6.** As in Fig 1. (top), Fig. 3 (middle), and Fig. 5 (bottom) of main article, but using HadCRUT4 in place of GISTEMP in the analysis.

**Figure S7.** As in Fig. 1 (top), Fig. 3 (middle), and Fig. 5 (bottom) of main article, but using only AIE simulations.

**Figure S8.** NH (left) and Global (right) mean CMIP5 surface temperatures. Raw annual mean of anthropogenic-only experiments are shown (blue) along with smoothed, extended series before (red) and after (black) correction for revised post-1986 anthropogenic forcing as per ref 12.

**Figure S9.** NH mean CMIP5 multimodel mean natural forced response (based on subtraction of anthropogenic-only from all-forcing response).

**
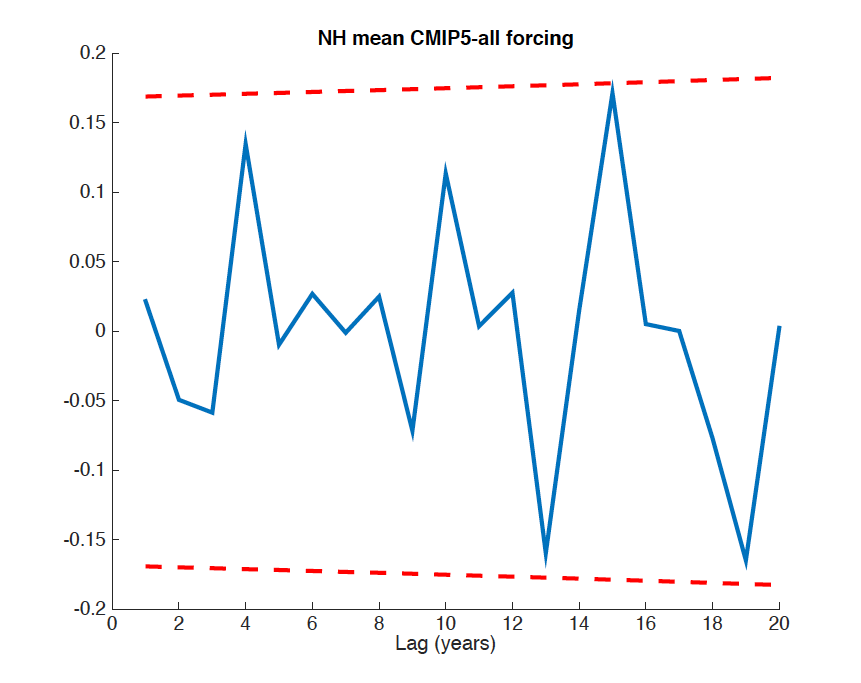

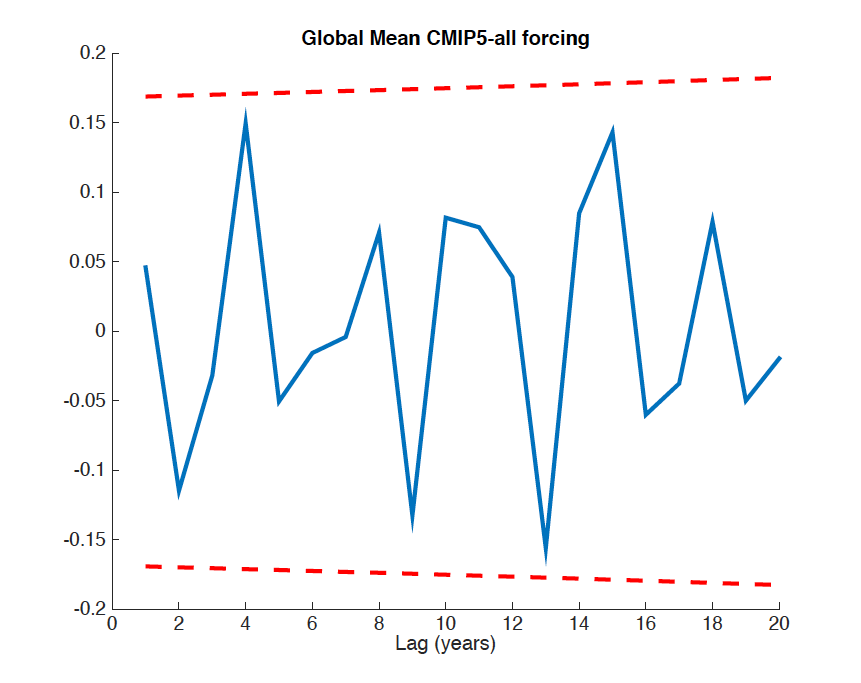
**

**
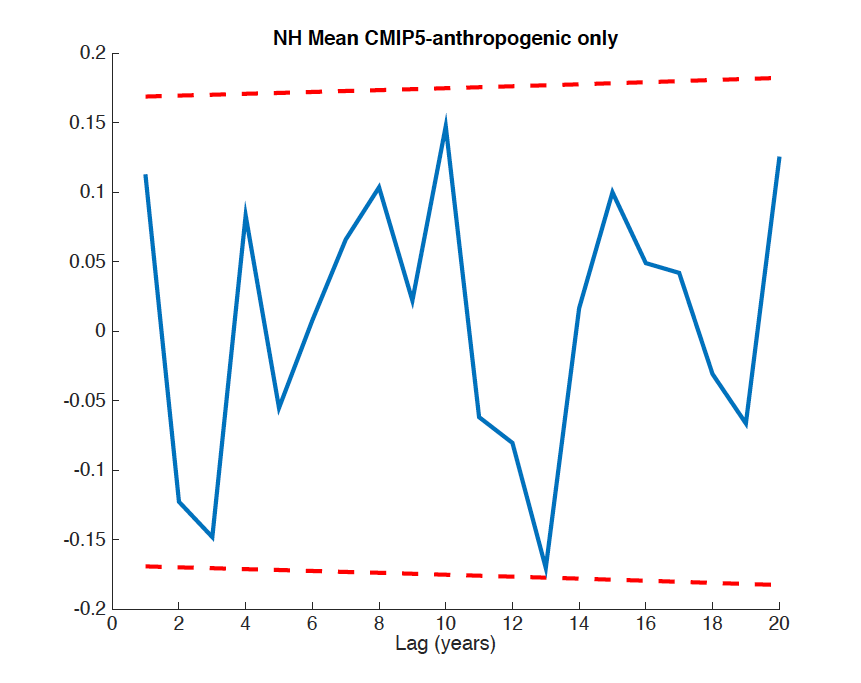

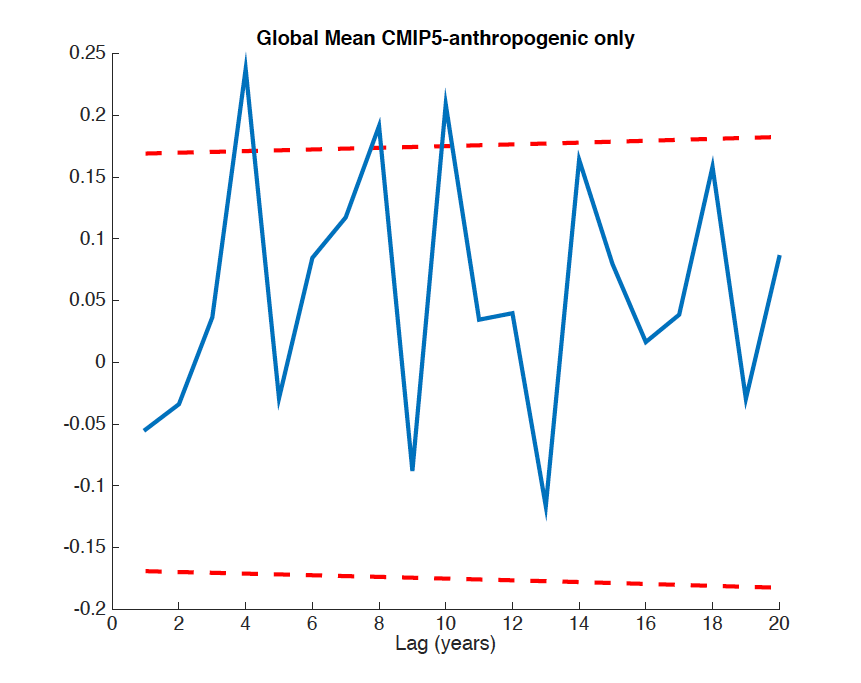
**

**Figure S10.** Autocorrelation (blue) for innovation series from statistical model fits for NH (left) and Global (right) mean temperature residuals. Results are shown for both (top) all-forcing case (where residual represents internal variability only) and (bottom) anthropogenic-only forcing case (where residual represents total natural variability). The dashed red curves indicate the two-sided 95% significance levels.
